# Supplementary material for: HIVprotI: an integrated web based platform for prediction and design of HIV proteins inhibitors
Source: J Cheminform. 2018 Mar 9;10:12. doi: 10.1186/s13321-018-0266-y (PMC5845081; doi:10.1186/s13321-018-0266-y)
Supplement: Supplementary file 2 — Additional file 2. Source code of HIVProtI web server. [file 13321_2018_266_MOESM2_ESM.zip › HIVprotI_Source-code/hivprotI/weka-3-6-6/doc/weka/core/xml/package-frame.html]

weka.core.xml


weka.core.xml

Classes 
  
KOML
  
MethodHandler
  
PropertyHandler
  
SerialUIDChanger
  
XMLBasicSerialization
  
XMLDocument
  
XMLInstances
  
XMLOptions
  
XMLSerialization
  
XMLSerializationMethodHandler
  
XStream
